# Mulberry Leaf Polyphenol Extract and Rutin Induces Autophagy Regulated by p53 in Human Hepatoma HepG2 Cells

**DOI:** 10.3390/ph14121310

**Published:** 2021-12-15

**Authors:** Meng-Hsun Yu, Ming-Chang Tsai, Chi-Chih Wang, Sheng-Wen Wu, Ya-Ju Chang, Cheng-Hsun Wu, Chau-Jong Wang

**Affiliations:** 1 Institute of Medicine, Chung Shan Medical University, Taichung 402, Taiwan; ya780522@gmail.com (M.-H.Y.); tsaimc1110@gmail.com (M.-C.T.); bananaudwang@gmail.com (C.-C.W.); y07172@msn.com (Y.-J.C.); 2 Department of Health Industry Technology Management, Chung Shan Medical University, Taichung 402, Taiwan; 3 School of Medicine, Chung Shan Medical University, Taichung 402, Taiwan; s41111.tw@yahoo.com.tw; 4 Division of Gastroenterology and Hepatology, Department of Internal Medicine, Chung Shan Medical University Hospital, Taichung 402, Taiwan; 5 Division of Nephrology, Department of Medicine, Chung Shan Medical University Hospital, Taichung 402, Taiwan; 6 Department of Anatomy, China Medical University, Taichung 406, Taiwan; 7 Department of Biochemistry, China Medical University, Taichung 406, Taiwan; 8 Department of Medical Research, Chung Shan Medical University Hospital, Taichung 402, Taiwan

**Keywords:** mulberry leaf polyphenol extract, rutin, human hepatoma HepG2 cells, autophagy, p53

## Abstract

The edible leaves of the mulberry (*Morus alba* L.) plant are used worldwide. They contain abundant polyphenolic compounds with strong anticancer properties. We previously revealed that apoptosis was mediated in p53-negative Hep3B cells, and mulberry leaf polyphenol extract (MLPE) induced autophagy in p53-transfected Hep3B cells. However, how this autophagy is induced by p53 in human hepatoma HepG2 (p53 wild type) cells remains unclear. In the current study, MLPE induced autophagy, as demonstrated by enhanced acidic vesicular organelle staining, by upregulating beclin-1, increasing LC3-II conversion, and phosphorylating AMPK. In HepG2 cells, these processes were associated with p53. Western blot also revealed phosphatidylinositol-3 kinase (PI3K), p-AKT, and fatty acid synthase (FASN) suppression in MLPE-treated cells. Moreover, treatment with the p53 inhibitor pifithrin-α (PFT-α) inhibited autophagy and increased apoptotic response in MLPE-treated HepG2 cells. PFT-α treatment also reversed MLPE-induced PI3K, p-AKT, and FASN suppression. Thus, co-treatment with MLPE and PFT-α significantly increased caspase-3, caspase-8, and cytochrome c release, indicating that p53 deficiency caused the apoptosis. In addition, rutin, a bioactive polyphenol in MLPE, may affect autophagy in HepG2 cells. This study demonstrates that MLPE is a potential anticancer agent targeting autophagy and apoptosis in cells with p53 status. Moreover, this work provides insight into the mechanism of p53 action in MLPE-induced cytotoxicity in hepatocellular carcinoma.

## 1. Introduction

Hepatocellular carcinoma (HCC) is a major cancer worldwide [1]. HCC risk factors include human immunodeficiency virus, hepatitis B virus, alcoholic liver disease, and nonalcoholic fatty liver disease [2]. Treatment options include surgical resection, chemotherapy, and radiotherapy [3]. Recent studies have shown that surgical resection and transplantation remain the curative standard of care for early-stage patients. Moreover, the effects of immunotherapy and personalized biomolecule signatures on improvement of HCC survival rate provide a stronger strategy to tackle this disease [4]. However, the 5-year survival rate is limited by high recurrence rates, highlighting the need for adjuvant therapies with low toxicity in patients with advanced HCC.

Mulberry (*Morus alba* L.) is an edible plant used to raise silkworms, and it is broadly distributed in tropical and temperate regions [5]. The roots, bark, leaves, and fruit of the mulberry are used in folk medicines and considered to be highly nutritious and anti-oxidative [6]. Mulberry leaf contains high levels of polyphenolic compounds, such as chlorogenic acid, rutin, isoquercitrin, quercetin, astragalin, and kaempferol [7]. Mulberry leaf extracts reportedly relieve obesity-related inflammation, help to attenuate type 2 diabetes, and inhibit atherosclerosis [8]. The mulberry leaf polyphenol extract (MLPE) has been reported to have numerous beneficial functions, such as those affecting antioxidants and free radicals [9]. The anticancer properties of MLPE have been reported for various types of cancer cells, including liver, breast, colon, and lung cancer cells [10]. However, MLPE’s anti-HCC mechanism remains poorly understood.

Under stress, the cell-survival pathway of autophagy is upregulated. Nutritional deprivation, hypoxia, infection, and the accumulation of damaged proteins can upregulate autophagy, during which cytoplasmic components are engulfed by autophagosomes; consequently, a microtubule-associated protein light chain 3 (LC3)-I with a cytosolic form conjugates to phosphatidylethanolamine, generating LC3-II [11]. However, severe autophagy may lead to a prodeath response. Autophagy and apoptosis might be associated, implying that the two processes have common regulators [12]. Beclin-1, a key molecule in autophagy, exhibits a structural similarity to the Bcl-2 homology 3 domain [13]. Common transcription factors are involved in the regulation of both autophagy and apoptosis. These factors include PI3K, tumor protein p53, c-Jun N-terminal kinase, and nuclear factor-kappaB (NF-kB) [14]. Beclin-1 and p53 are thought to link apoptosis with autophagy. Studies have discovered a p53 mutation in various human cancers, and such mutation is common in primary liver cancers. In 50% of human cancers, the p53 suppressor gene is inactivated, and patients with such gene inactivation have shorter survival rates than patients with wild p53 in liver carcinoma do [15].

We previously reported that apoptosis was induced by MLPE in Hep3B cells via a pathway independent of p53; MLPE also induced autophagy in Hep3B cells transfected with p53 [16]. We suspected p53 involvement in autophagy regulation. We therefore investigated the pathways that MLPE regulates to induce p53-dependent autophagy in human HepG2 (p53-positive) cells and p53-independent apoptosis to extend the aforementioned findings.

## 2. Results

### 2.1. HPLC Characterization of MLPE Polyphenols

**Figure 1:**
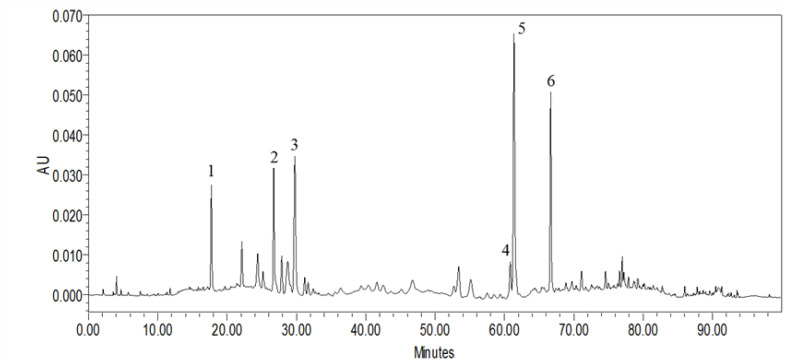
Composition and characterization of phenolic compounds contained in MLPE. The HPLC chromatogram and absorbance at 280 nm for polyphenols were monitored for MLPE.

The quantities of each polyphenol in the MLPE are presented in Figure 1. The phenolic compounds in dried mulberry leaves were protocatechuic acid, chlorogenic acid, cryptochlorogenic acid, nicotiflorin, rutin, and astragalin compounds, with respective contents of 6.25, 12.06, 19.7, 8.34, 45.35, and 24.67 μg/mg. The most abundant polyphenol in MLPE was rutin, presented in Table 1.

### 2.2. MLPE Cytotoxicity to Various Cell Lines

**Figure 2:**
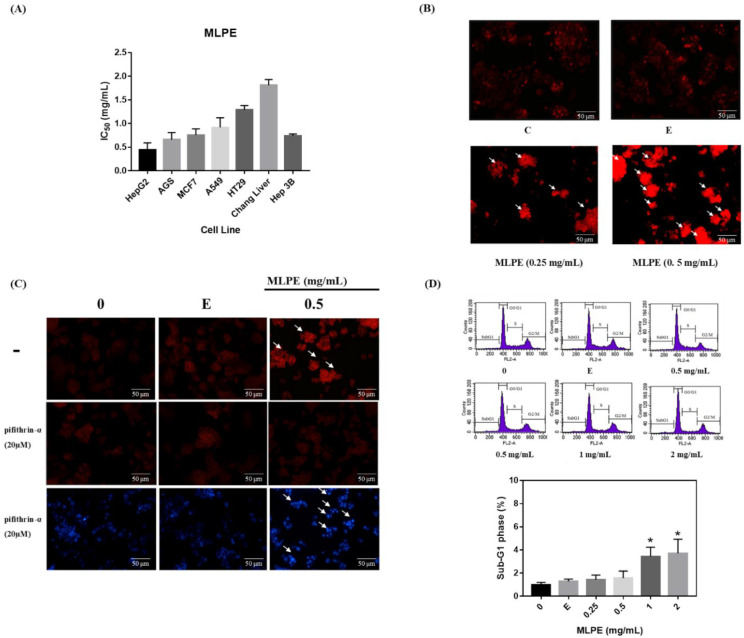
Autophagy-dependent p53 effects of MLPE on HepG2 cells. (**A**) IC50 of MLPE on the viability in different cell lines. (**B**) HepG2 cells were treated with 0, 0.25, 0.5 mg/mL of MLPE and solvent for 24 h and subjected to AVO staining. The arrow indicated AVO cells. AVOs values were calculated as the percentage of AVO cells relative to the total number of cells in each random field. Results were statistically analyzed with Student’s t-test. Magnification: 400×. (**C**) HepG2 cells were pretreated with PFTα for 6 h and treated with 0, 0.5 mg/mL of MLPE and solvent for 24 h and subjected to DAPI/AO staining. (**D**) Apoptosis effects of MLPE on HepG2 cells. HepG2 cells were treated with 0, 0.25, 0.5, 1, 2 mg/mL of MLPE and solvent (0.05% EtOH) for 24 h and subjected to flow cytometric analysis after PI staining. The figure shows a representative staining profile for 10,000 cells per experiment. Sub G1 was defined as apoptotic cells and represents the average of three independent experiments ±SD, *n* = 3. *, *p* < 0.05 compared with E. Magnification: 100×. The figure shows a representative staining profile for 8000 cells per experiment. C, control; E, ethanol.

To explore the antitumor activity of MLPE in various tumor cells, the viabilities of HepG2, AGS, MCF-7, A549, HT29, Chang liver, and Hep3B cells were measured through the MTT assay. The half-maximal inhibitory concentration (IC50) values of these cells were 0.44, 0.66, 0.75, 0.92, 1.29, 1.81, and 0.74 mg/mL, respectively. Compared with the other cancer cells, the HepG2 cells had the lowest IC50 after MLPE treatment (Figure 2A). Therefore, we investigated the anticancer mechanism of MLPE by using HeG2 cells.

### 2.3. Regulation of MLPE-Induced Autophagy in HepG2 Cells by p53

Plant-derived compounds exert anticancer effects by inducing autophagy in cancer cells [17]^.^ The morphological characteristics indicating autophagy were detected through acidic vesicular organelle (AVO) fluorescence staining. Therefore, we tested whether MLPE induced autophagy in HepG2 cells. After MLPE treatment (0, 0.25, and 0.5 mg/mL), AO staining and fluorescence microscopy were performed on the cells. AVO formation after 24 h increased in HepG2 cells treated with 0.5 mg/mL MLPE (Figure 2B). PFT-α could prevent cell apoptosis in a p53-independent manner [18]. To evaluate whether autophagy induction in HepG2 cells is associated with p53 activation, the effects of the p53 inhibitor PFT-α on MLPE-treated cells were assessed through AVO staining. AVO formation was reduced in cells treated with MLPE (Figure 2C). Subsequently, we evaluated apoptosis expression in HepG2 cells co-treated with MLPE and PFT-α. The DAPI stain revealed significant apoptotic nuclear chromatin condensation in these cells (Figure 2C). To determine whether MLPE induced apoptosis in HepG2 cells, we evaluated the cell cycle sub-G1 ratios, which exhibited a slight increase following 0.5 mg/mL MLPE treatment (Figure 2D). These data indicate that MLPE has little influence on apoptotic HepG2 cells.

### 2.4. Effect of MLPE on HepG2 Autophagy

**Figure 3:**
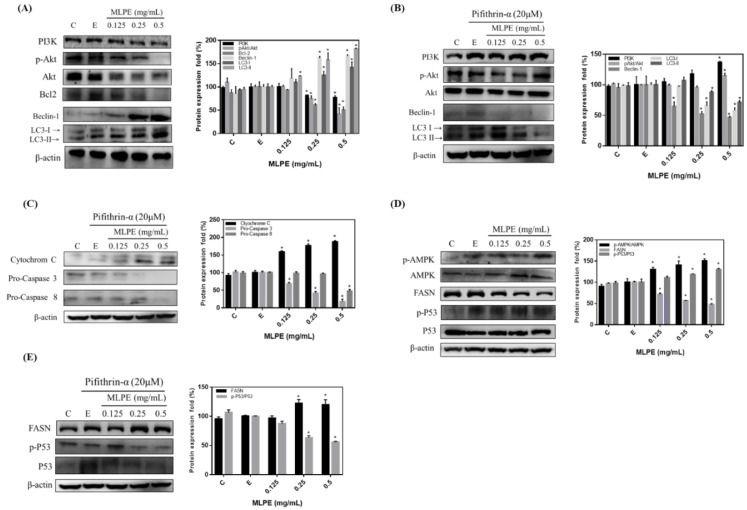
Immunoblot analysis of the autophagosome induction of autophagy in HepG2. Cultured cells were treated with 0, 0.125, 0.25, 0.5 mg/mL of MLPE, PFT α for 6 h and treated with various concentrations of MLPE and solvent (0.05% EtOH) for 24 h, and whole-cell extracts were prepared as described in Materials and Methods. Equal amounts of total proteins were loaded in each lane of SDS-polyacrylamide gel (protein concentration is 50 μg/μL). Western hybridization was performed with antibodies against (**A**,**B**) PI3K, p-Akt/Akt, Bcl-2, Beclin-1, LC3-I, and LC3-II. (**C**) Cytochrome C, pro-caspase 3, and pro-caspase 8. (**D**,**E**) p-AMPK/AMPK, FASN, and p-P53/P53. Western blot analysis of β-actin was used as an internal control and represents the average of three independent experiments ±SD, *n* = 3. *, *p* < 0.05 compared with (**E**).

PI3K, AKT, and p-AKT expression were determined through Western blot. MLPE suppressed PI3K, AKT, and p-AKT activation in HepG2 cells (Figure 3A). Moreover, the MLPE-induced suppression of PI3K, AKT, and p-AKT was rescued by PFT-α (Figure 3B). To further investigate whether autophagy was regulated by MLPE treatment, we analyzed the levels of LC3-II and beclin-1, which indicate autophagy. As presented in Figure 3A, MLPE treatment at 0.25 and 0.5 mg/mL induced dominant LC3-II formation and beclin-1 activation, respectively, in HepG2 cells when Bcl-2 expression was suppressed. Furthermore, PFT-α blocked LC3-II and beclin-1 protein expression significantly (Figure 3B).

To determine whether the MLPE-induced cytotoxicity in PFT-α-treated HepG2 cells was due to apoptosis, Western blot was performed after treatment with MLPE at various concentrations for 24 h. Cytochrome c protein levels significantly increased in the MLPE–PFT-α treatment group. However, the expression of both pro-caspase-3 and pro-caspase-8 significantly decreased in the cells treated with both MLPE and PFT-α (Figure 3C). We also investigated whether p53-regulated AMPK/FASN expression is associated with MLPE-induced autophagy. Increased AMPK and p53 phosphorylation was observed, and FASN expression was reduced after treatment with MLPE at 0.25 and 0.5 mg/mL (Figure 3D). In addition, FASN expression was increased in the cells treated with MLPE and PFT-α (Figure 3E). These results indicate that autophagy inhibition by the p53 inhibitor PFT-α led to MLPE-induced apoptosis, and that p53 is a key regulator.

### 2.5. Rutin Mediation of Autophagy in MLPE-Treated HepG2 Cells

**Figure 4:**
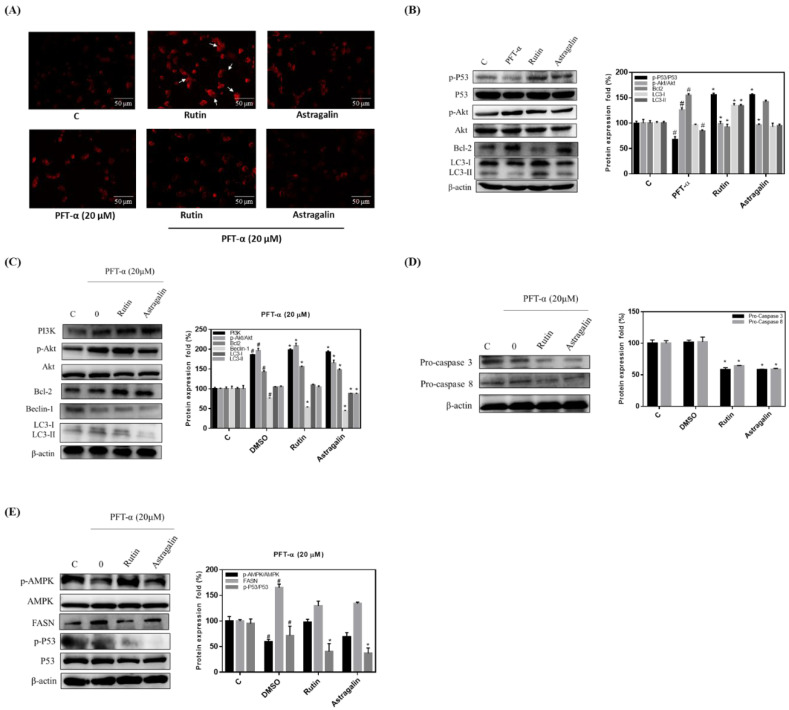
Rutin induced autophagy-dependent p53 in HepG2 cells. (**A**) HepG2 cells were treated with PFT α for 6 h and treated with 1 mM rutin, 0.1 mM of astragalin for 24 h and subjected to AVO staining. The arrow indicated AVO cells. AVOs values were calculated as the percentage of AVOs cells relative to the total number of cells in each random field. Results were statistically analyzed with Student’s *t*-test. Magnification: 400×. (**B**) Cultured cells were treated with 1 mM rutin, 0.1 mM of astragalin, and PFT-α for 24 h, and whole-cell extracts were prepared as described in Materials and Methods. Equal amounts of total proteins were loaded in each lane of SDS-polyacrylamide gel (protein concentration is 50 μg/μL). Western hybridization was performed with antibodies against p-P53/P53, pAkt/Akt, Bcl-2, LC3-I, and LC3-II. (**C**) PI3K, pAkt/Akt, Bcl-2, Beclin-1, LC3-I, and LC3II. (**D**) Pro-caspase 3, and pro-caspase 8. (**E**) pAMPK/AMPK, FASN, and p-P53/P53. Western blot analysis of β-actin was used as an internal control and represents the average of three independent experiments ±SD, *n* = 3. #, *p* < 0.05 compared with C. *, *p* < 0.05 compared PFT α.

Rutin and astragalin are phenolic components of MLPE with various reported biochemical activities [19]. Through AVO staining, we investigated the phenolic components of both rutin and astragalin in regulating autophagy. AVO formation was increased in rutin-treated cells but not in astragalin-treated cells (Figure 4A). PFT-α significantly blocked this AVO formation in rutin-treated cells (Figure 4A). Moreover, p53 phosphorylation and LC3-II accumulation were markedly greater, and Bcl-2 expression and AKT phosphorylation were lower in rutin-treated HepG2 cells than in astragalin-treated cells (Figure 4B). After co-treatment with PFT-α and rutin or astragalin, the Western blot indicated increased PI3K/AKT and Bcl-2. By contrast, LC3-I/LC3-II and beclin-1 were reduced in HepG2 cells (Figure 4C). Moreover, pro-caspase-3 and pro-caspase-8 decreased in rutin-treated and astragalin-treated cells that were also treated with PFT-α (Figure 4D). Astragalin-induced autophagy was not significant in HepG2 cells. These results demonstrate that MLPE triggers autophagy, and that rutin is the bioactive compound of MLPE.

## 3. Discussion

**Figure 5:**
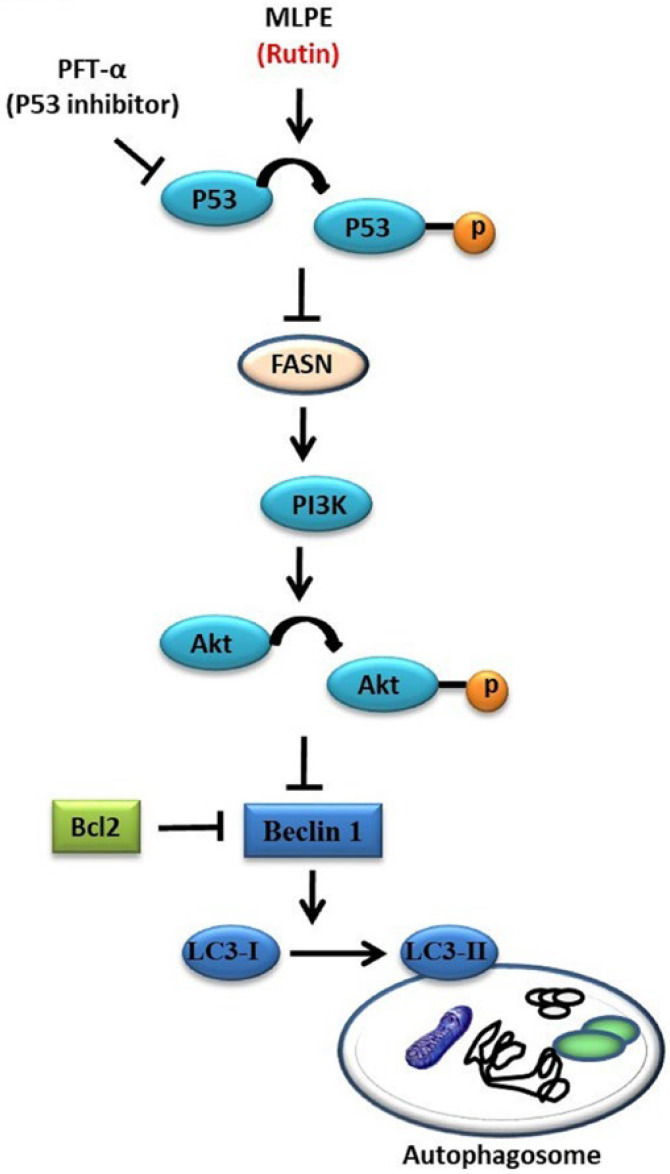
Schematic diagram illustrating the p53-dependent autophagy of HepG2 cell treated with MLPE or rutin.

Mulberry leaves contain polyphenols and are used in Asian medicinal formulas. MLPE reportedly benefits human health [6,8]. We previously demonstrated that MLPE induced apoptosis and autophagy in p53-negative and p53-positive (p53-transfected) Hep3B cells, respectively [16]. However, the p53 signaling underlying the autophagy and apoptosis crosstalk has yet to be described. Here, we investigated the anticancer effects of MLPE on p53-positive HepG2 cells. Our results indicate that autophagy is induced in p53-positive HepG2 cells by MLPE treatment, and apoptosis occurs in a p53-indepedent manner in HepG2 cells. Moreover, MLPE increased LC3-II conversion and upregulated beclin-1, leading to autophagy accompanied by PI3K, p-AKT, and Bcl-2 down-regulation. PFT-α, a p53 inhibitor, suppressed autophagy and enhanced apoptosis-related protein expression (Figure 5). This mechanism may explain how autophagy inhibition can sensitize tumor cells to apoptosis.

How autophagy can be used in potential cancer therapies is controversial. Strategies for autophagy mediation in tumor cells can be prosurvival or prodeath [12]. Specifically, autophagy has a tumor-suppressive function in apoptosis-resistant tumor cells. A previous study reported that autophagy may be caused by some therapeutic drugs; the use of such drugs can counteract drug resistance in cancer cells [20]. Moreover, autophagy may be regarded as a prosurvival mechanism that helps tumor cells to respond to metabolic stress and survive chemotherapy. Beclin-1 cannot trigger autophagy while bound to Bcl-2. Beclin-1 is released by proapoptotic Bcl-2 homology 3 proteins from Bcl-2 to induce autophagy [21].

In this study, MLPE induced autophagy by increasing LC3-II (autophagosome formation) and beclin-1 levels and reducing Bcl-2 expression (Figure 3). On the other hand, the previous study had shown that autophagy activation is related to the down-regulation of p62 levels [22,23]. It was also reported that using the sinensetin (SIN) significantly increased the ratio of LC3-II/I, beclin-1, and decreased p62 levels in HepG2 cells [24]. Additionally, autophagy inhibition by the p53 inhibitor PFT-α enhanced MLPE-induced apoptosis by activating cytochrome c, caspase-3, and caspase-8 in HeG2 cells (Figure 3). Moreover, co-treatment with PFT-α and SIN resulted in up-regulation of p62, PARP, and caspase-3 proteins and down-regulation of p-AMPK and LC3B-II expression compared with SIN treatment alone in HepG2 cells. In the HepG2 cells treated with SIN, blocking p53 translocation by PFT-α induced apoptosis instead of autophagy [24]. Therefore, p53 deficiency changed the HepG2 cell response to MLPE-induced apoptosis when autophagy was suppressed. Our data are inconsistent with the assumption of a prosurvival pathway of autophagy that promotes cancer growth. However, the molecular mechanism that connects autophagy and apoptosis is poorly understood. Fitzwalter et al. demonstrated that increased FOXO3a expression upon autophagy inhibition caused apoptosis sensitization [25]. Other mechanisms may include the accumulation of polyphenols in the mitochondria, thereby regulating the mitochondrial electron transport chain and Bcl-2 protein family, leading to mitochondrial apoptosis, even under p53-deficient conditions [26]. We demonstrated that MLPE enhanced cytochrome c release, which was followed by caspase-3 and caspase-8 activation in HepG2 cells treated with PFT-α, indicating mitochondrial dysregulation (Figure 3C).

The tumor suppressor p53 is crucial to numerous cellular responses, to stress signals, and triggers responses such as senescence, apoptosis, and cell cycle arrest [27]. In one study, p53 suppression increased autophagy in various types of cells. Lee et al. demonstrated that luteolin induced autophagy only in p53-null Hep3B cells [28]. By contrast, Wei et al. indicated that XingNaoJing inhibits autophagy by suppressing the p53 signaling pathway in PC12 cells [29]. Similarly, our results demonstrate that MLPE induces cell apoptosis by suppressing autophagy under p53-deficient conditions. The mammalian target of rapamycin (mTOR) is crucial in autophagy induction in various pathways. Specifically, active p53/AMPK inhibits mTOR activity, thereby inducing autophagy. The inhibition of the PI3K/AKT pathway also leads to autophagy activation through mTOR inhibition [30]. In a previous study, cell proliferation and cell survival were increased by activating the PI3K and AKT signaling pathways [31]. Relevant studies have indicated that AMPK acts as a metabolic tumor suppressor. AMPK activation reprograms cellular metabolism and regulates various targets downstream, including FASN, p53, and mTORC1. De novo free fatty acid synthesis requires the downstream target FASN, which is highly expressed in various cancers. FASN inhibition reportedly causes cancer cell apoptosis [32]. FASN inhibition induced both autophagy and endoplasmic reticulum stress in human breast cancer cells [33]. Consistent with these observations, we demonstrated that p53 inactivation induced by MLPE treatment negatively regulated autophagy via the AMPK/FASN signaling pathway. It then downregulated PI3K/AKT expression, LC3-II conversion, and beclin-1 release from Bcl-2.

The abundant polyphenols in mulberry leaves, including protocatechuic acid, chlorogenic acid, nicotiflorin, rutin, and astragalin, have different bioactivities and functions. Rutin is the most abundant bioactive flavonoid in MLPE (Figure 1). It has diverse pharmacological properties, including antioxidative and anticancer activities. Nabavi et al. reported that polyphenols can regulate autophagy [34]. Another study reported that rutin induced autophagy in oral (CA9-22) and lung (A549) cancer cells [35]. Similarly, our findings indicate that rutin in MLPE regulates autophagy in HepG2 cells by influencing p53 signaling (Figure 5).

## 4. Materials and Methods

### 4.1. Chemicals and Reagents

This study used acridine orange (AO), 4′,6-diamidino-2-phenylindole (DAPI), and pifithrin-α (PFT-α) (Sigma-Aldrich, St. Louis, MO, USA); anti-p53, anti-caspase-8, anti-caspase-3, anti-β-actin, anti-AKT, anti-PI3K, anti–fatty acid synthase (FASN), anti-AMPK, anti-LC3, and anti-beclin-1 antibodies (1:1000 dilution; Cell Signaling Technology, Danvers, MA, USA); and anti-mouse and anti-rabbit horseradish peroxidase–conjugated secondary antibodies (1:5000; Santa Cruz, CA, USA).

### 4.2. MLPE Preparation and High-Performance Liquid Chromatography

Leaves from mulberry (*M. alba*) plants were acquired from Miaoli District Agricultural Research and Extension Station in Miaoli County, Taiwan. Dried mulberry leaves were used to prepare MLPE. In brief, 100 g of mulberry leaves was combined with 300 mL of methanol and heated at 50 °C for 3 h. Subsequently, the filtrate was collected and evaporated under vacuum, then kept at −20 °C until use. A solution of the mulberry extract was created in 500 mL of ddH_2_O and then added to 200 mL of n-hexane and left overnight to collect the water layer. Next, 180 mL of ethyl acetate was used to extract the polyphenols for high-performance liquid chromatography (HPLC; Hitachi, Danbury, CT, USA). The powders were resuspended in 0.05% EtOH, filtered with a sterile 0.22 μm filter, and then used in cell culture.

A Waters system (including the 2998 photodiode array detector, 600 HPLC controller and pump, and four-channel In-Line Degasser-AF) was used for HPLC. A Mightysil RP-18 GP (particle size: 5 μm, i.d.: 250 × 4.6 mm) at room temperature was employed as the separating column. Formic acid in water (pH: 2.5), acetonitrile, and methanol—respectively termed solutions A, B, and C—formed the mobile phase. The following gradient elution program was used for HPLC with a flow rate of 1.0 mL/min, injection volume of 20 μL, and ultraviolet detection range of 210–400 nm: at minute 0, 100% A; at minute 5, 90% A, 0% B; at minute 15, 85% A, 3% B; at minute 20, 85% A, 5% B; at minute 30, 85% A, 8% B; at minute 50, 85% A, 15% B; at minute 65, 75% A, 25% B; at minute 70, 70% A, 30% B; at minute 80, 50% A, 50% B; at minute 85, 100% B; and at minute 100, 100% B.

### 4.3. Cell Line Culture

The Bioresource Collection and Research Center (Food Industry Research and Development Institute, Hsinchu, Taiwan) provided all cell lines used in the present study. In Dulbecco’s modified Eagle’s medium were kept human hepatoma HepG2 cells, HCC Hep3B cells, and human breast carcinoma cells (MCF-7). Chang liver cells (normal liver cells) were kept in Eagle’s basal medium. In F-12 nutrient mixture medium were kept human colonic adenocarcinoma cells (HT29) and gastric adenocarcinoma cells (AGS). Medium supplemented with 2 mM glutamine, 1% antibiotics (100 μg/mL streptomycin and 100 U/mL penicillin), and 10% fetal bovine serum was used to culture all cell lines under 5% CO_2_ at 37 °C.

### 4.4. Cell Viability Assay

Each cell line (density: 7 × 10^4^ cells) was seeded in 24-well plates and underwent 24 h of treatment with MLPE at various concentrations (dissolved in 50% ethanol). After the addition of 3-(4,5-dimethylthiazol-2-yl)-2,5-diphenyltetrazolium bromide (MTT) and 3 h of incubation, the MTT was converted into formazan crystals. The purple-blue formazan crystals were then washed with PBS. After dissolution in 1 mL of DMSO, absorbance at 563 nm was measured.

### 4.5. DAPI and Acidic Vesicular Organelle Fluorescence Staining

In six-well plates, HepG2 cells (density: 1 × 10^5^ cells) were seeded and treated with pretreated PFT-α or 0, 0.25, or 0.5 mg/mL of MLPE for 6 h and then co-treated with MLPE for 24 h. At 37 °C, the cells were exposed to DAPI (4′,6-diamidino-2 phenylindole, 1 μL/mL) for 5 min and then subsequently to AO solution (5 μg/mL) for 15 min. After the HepG2 cells were incubated with dye, they were suspended in 1× PBS and analyzed through fluorescence microscopy at 400× magnification (Nikon DIAPHOT-300, Tokyo, Japan).

### 4.6. Flow Cytometry for Cell Cycle Analysis

In six-well plates, the HepG2 cells (density: 2 × 10^5^ cells) were seeded and treated for 24 h with 0, 0.25, 0.5, 1, or 2 mg/mL MLPE. They were subsequently collected and fixed in ethanol (70%). After 24 h of incubation at −20 °C, the samples underwent centrifugation for 5 min at 1200 rpm. The resulting pellets were then resuspended in 40 μg/mL PI solution, 100 μg/mL RNase, and 1 × PBS and incubated in the dark at 37 °C for 15 min. Flow cytometry (BD Biosciences, Bedford, MA, USA) was conducted to analyze the samples. C6 software (BD Biosciences) was used for cell cycle distribution analysis.

### 4.7. Protein Extraction and Western Blot

Western blot was performed after the HepG2 cells had been successively treated with PFT-α and MLPE for 6 and 24 h, respectively, to determine how MLPE affected autophagy and markers for apoptosis. We washed the samples in ice-cold PBS. A solution containing the following components was then used to lyse the samples on ice: a protease inhibitor cocktail (Roche, Indianapolis, IN, USA), 150 mM NaCl, 20 mM Tris-HCl (pH 7.5), 1 mM EDTA, Nonidet P-40 0.5% (*v*/*v*), 1 mM DTT, and 0.5 mM PMSF. We employed a Bradford protein assay kit to measure protein concentrations. Next, 12% sodium dodecyl sulphate–polyacrylamide gel electrophoresis was used to separate 30 μg protein samples. We then moved the protein to PVDF membranes (Roche), which were then blocked with Tris-buffered saline–Tween20 (TSBT; Sigma-Aldrich) containing 5% blocking solution. Appropriate primary antibodies and secondary horseradish peroxidase–conjugated antibodies were then used for incubation (GE Healthcare, Little Chalfont, Buckinghamshire, UK). We washed the membranes in TBST extensively and used enhanced chemiluminescence to detect protein bands (Amersham Pharmacia Biotech, UK).

### 4.8. Statistical Analysis

All experiments expressed as the mean ± SD. Statistical analysis was performed using Students’ *t*-test and were performed at least three times. The results indicated a statistically significant difference at *p* values < 0.05.

## 5. Conclusions

Our results demonstrate that MLPE induces autophagy by activating the p53 pathway in human HepG2 cells. The consequent inhibition of the p53 signaling induced by PFT-α enabled the transition from autophagy to apoptosis. Further research is required to determine whether MLPE can be used for cancer therapy.

## Figures and Tables

**Table 1:** The isolated phenolic compounds (numbered peaks) identified in MLPE by LC-MS/MS.

Peak No.	RT (min)	[M + H]^+^ (*m*/*z*)	UV Band (nm)	Compounds Name	Concentration (μg/mg)
1	17.72	153.1 [M − H]^−^	259.7 (max), 291	Protocatechuic acid	6.25 ± 0.33
2	26.73	355.7 [M + H]^+^	241.9, 326.3 (max)	Chlorogenic acid	12.06 ± 0.41
3	29.77	355.7 [M + H]^+^	238.4, 323.9 (max)	Cryptochlorogenic acid	19.70 ± 1.68
4	60.84	595.9 [M + H]^+^	265.6 (max), 346.6	Nicotiflorin	8.34 ± 0.31
5	61.38	609.2 [M − H]^−^	256.1 (max), 355.0	Rutin	43.35 ± 0.91
6	66.67	449.7 [M + H]^+^	265.6 (max), 346.6	Astragalin	24.67 ± 0.25

## Data Availability

Data is contained within the article.
